# Investigating the physiology of viable but non-culturable bacteria by microfluidics and time-lapse microscopy

**DOI:** 10.1186/s12915-017-0465-4

**Published:** 2017-12-21

**Authors:** Rosemary A. Bamford, Ashley Smith, Jeremy Metz, Georgina Glover, Richard W. Titball, Stefano Pagliara

**Affiliations:** 1 Biosciences, University of Exeter, Exeter, Devon EX4 4QD UK; 2 Living Systems Institute, University of Exeter, Exeter, Devon EX4 4QD UK

**Keywords:** Viable but non-culturable cells, Microfluidics, *Escherichia coli*, Persisters, Single-cell, Antibiotic tolerance, Non-growing cells

## Abstract

**Background:**

Clonal microbial populations often harbor rare phenotypic variants that are typically hidden within the majority of the remaining cells, but are crucial for the population’s resilience to external perturbations. Persister and viable but non-culturable (VBNC) cells are two important clonal bacterial subpopulations that can survive antibiotic treatment. Both persister and VBNC cells pose a serious threat to human health. However, unlike persister cells, which quickly resume growth following drug removal, VBNC cells can remain non-growing for prolonged periods of time, thus eluding detection via traditional microbiological assays. Therefore, understanding the molecular mechanisms underlying the formation of VBNC cells requires the characterization of the clonal population with single-cell resolution. A combination of microfluidics, time-lapse microscopy, and fluorescent reporter strains offers the perfect platform for investigating individual cells while manipulating their environment.

**Methods:**

Here, we report a novel single-cell approach to investigate VBNC cells. We perform drug treatment, bacterial culturing, and live/dead staining in series by using transcriptional reporter strains and novel adaptations to the mother machine technology. Since we track each cell throughout the experiment, we are able to quantify the size, morphology and fluorescence that each VBNC cell displayed before, during and after drug treatment.

**Results:**

We show that VBNC cells are not dead or dying cells but share similar phenotypic features with persister cells, suggesting a link between these two subpopulations, at least in the *Escherichia coli* strain under investigation. We strengthen this link by demonstrating that, before drug treatment, both persister and VBNC cells can be distinguished from the remainder of the population by their lower fluorescence when using a reporter strain for *tnaC*, encoding the leader peptide of the *tnaCAB* operon responsible for tryptophan metabolism.

**Conclusion:**

Our data demonstrates the suitability of our approach for studying the physiology of non-growing cells in response to external perturbations. Our approach will allow the identification of novel biomarkers for the isolation of VBNC and persister cells and will open new opportunities to map the detailed biochemical makeup of these clonal subpopulations.

**Electronic supplementary material:**

The online version of this article (doi:10.1186/s12915-017-0465-4) contains supplementary material, which is available to authorized users.

## Background

Clonal bacterial populations show cell-to-cell differences in physiological parameters, including responses to external perturbations [1]. For example, under drug treatment, the majority of a clonal bacterial population is susceptible to the drug, whereas at least two subpopulations – persister cells and viable but non-culturable (VBNC) cells – are able to survive high doses of antibiotics. Persister cells survive the antibiotic challenge and resume growth on nutrient-rich media shortly after removing the drug [2, 3], whereas VBNC cells survive the antibiotic challenge but may regrow only after a long and specific treatment [4]. Therefore, VBNC cells are difficult to detect and study via standard microbiology assays.

Persister and VBNC cells are in a transient state and are often, but not always [5], associated with dormancy [2, 6]. It has been suggested that they form stochastically as a result of fluctuations in gene and protein expression [6, 7]. However, deterministic components of persister and VBNC formation have also been documented [8–13]. Persister and VBNC cells pose a serious threat to human health since they may be recalcitrant to drug treatment [2], and thus can contribute to the relapse of diseases such as tuberculosis, cystic fibrosis-associated lung infections, candidiasis, cholera, septicemia, and gastroenteritis [4]. Furthermore, these subpopulations may constitute a reservoir for the development of antibiotic resistance mechanisms [14].

The recent advances in single-cell microfluidic analysis [15] have greatly enriched our understanding of persister cells, highlighting the contributions from toxin-antitoxin modules [6], the alarmone guanosine tetraphosphate pathway [7, 16, 17], efflux activity [18], the catalase-peroxidase enzyme [19], respiration [20], and protein production [21, 22]. However, single-cell microfluidic analysis has not yet been implemented to investigate VBNC cells due to the difficulty in distinguishing these cells from dead or dying cells, and the requirement to image and track thousands of individual cells. Nevertheless, VBNC cells constitute a major public health concern, since they have been observed in 51 human pathogens [3] and are difficult to eradicate via standard sterilization treatments such as heat, acid, ethanol, antibiotic or osmotic stress [23]. Therefore, there is an urgent to develop methodologies to study and combat bacteria in the VBNC state [4].

**Fig. 1:**
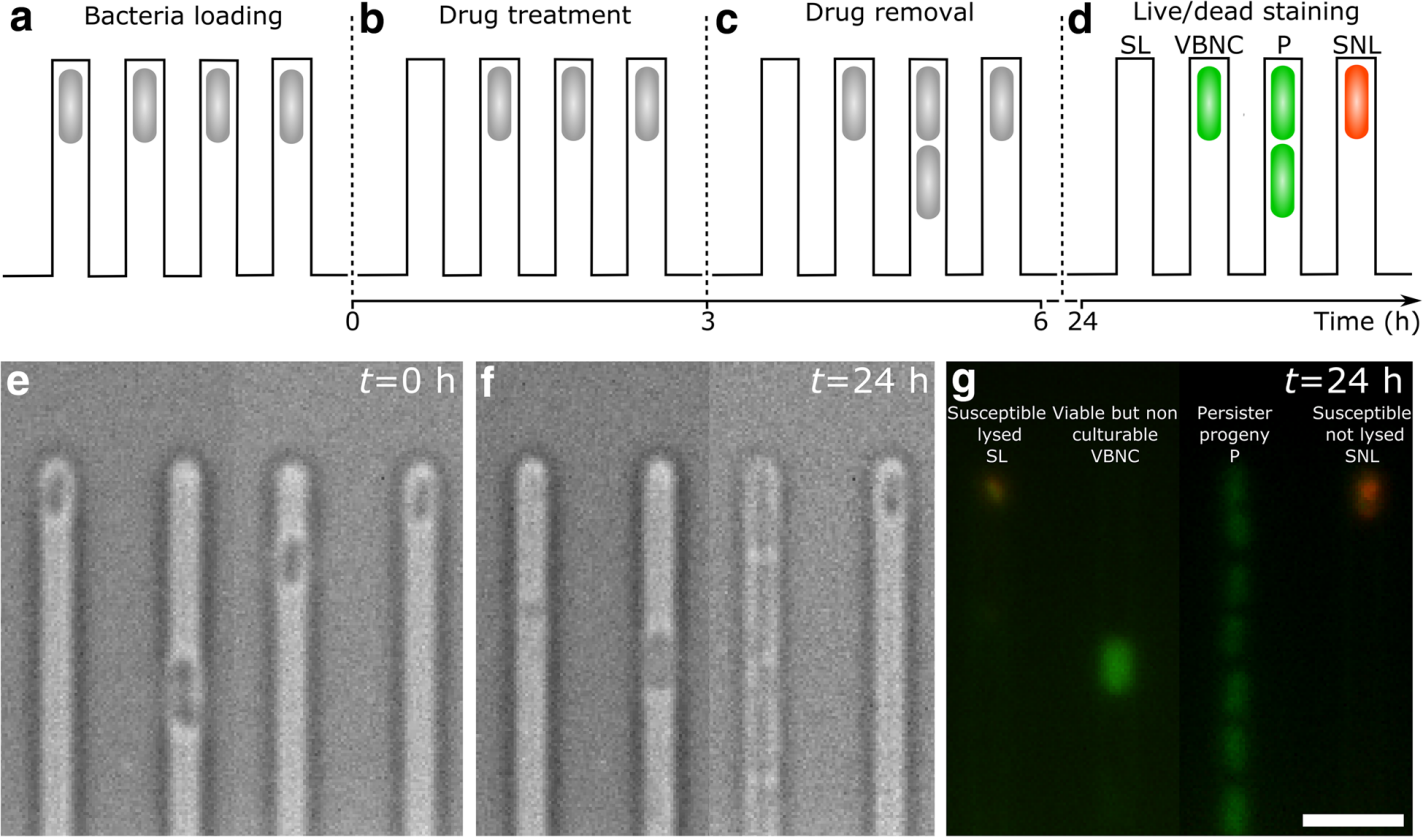
Single-cell approach to study viable but non-culturable (VBNC) cells. Schematic illustrating the steps carried out to distinguish VBNC cells from susceptible non-lysed (SNL), susceptible lysed (SL), and persister (P) cells. **a** A 2-μL aliquot of a stationary phase *E. coli* BW25113 culture was loaded in the lateral channels of the mother machine device. **b** Between *t* = 0 and *t* = 3 h, ampicillin was injected in the mother machine device at a concentration of 25 × the minimum inhibitory concentration in Lysogeny broth (LB) progressively lysing the majority of bacteria (SL cells, leftmost channel). **c** Between *t* = 3 and *t* = 24 h, LB was injected in the microfluidic device, P bacteria started to grow and eventually gave rise to progeny (third channel from the left). **d** At *t* = 24 h, a live/dead staining assay was performed by flowing in the microfluidic device SYTO9 and propidium iodide, distinguishing VBNC bacteria staining green (second channel from the left) from SNL bacteria staining red (rightmost channel). Representative bright-field (**e**–**f**) and fluorescence images (**g**) of the four phenotypes illustrated above, before (**e**) and after (**f**, **g**) drug treatment. The weak red staining in the channel containing the SL bacterium was due to cell debris barely visible in the corresponding bright-field image. Scale bar, 5 μm

Here, we introduce a novel single-cell approach allowing, for the first time, the investigation of the changes in size, morphology and promoter activity in VBNC cells responding to drug treatment (Fig. 1). We take advantage of the well-established mother machine device [24, 25], designed to investigate the growth of a large number of individual *E. coli* cells. This device is equipped with thousands of microfluidic channels with cross section comparable to the size of individual *E. coli* cells and connected to a large microfluidic chamber where the medium is continuously exchanged via pressure-driven microfluidics. In this paper, we use this technology to perform drug treatment, bacterial culturing, and live/dead staining in series, while imaging and tracking individual cells, thus permitting the identification of single VBNC cells alongside persister or susceptible cells. This new methodology allows us to obtain the following crucial information that will advance our understanding of VBNC cells. (1) We demonstrate that ampicillin-treated stationary phase *E. coli* cultures contain more VBNC than persister cells. (2) We show that, before drug treatment, VBNC cells exhibit cell length and levels of fluorescence for selected reporter strains similar to the ones measured in persister cells, supporting the hypothesis that these two phenotypes are part of a shared physiological continuum at least in the investigated *E. coli* strain [4]. (3) We demonstrate that, after drug treatment, VBNC cells are distinct from dead or dying cells and display fluorescence levels comparable to persister cells. (4) We identify the fluorescence of the *tnaC* reporter strain as a new biomarker for distinguishing persister and VBNC bacteria from the remainder of the population before drug treatment. Our novel single-cell approach will facilitate unraveling the molecular mechanisms underlying the formation of non-growing subpopulations and their capabilities to survive environmental changes. As such, our methodology represents a powerful tool for researchers investigating phenotypic or genotypic heterogeneity.

## Results

### Identification of VBNC cells

We chose to apply our novel single-cell approach to stationary phase *E. coli* cultures because the fraction of VBNC and persister cells in this growth phase is in the range 10^-3^–10^-1^ [18, 20, 26, 27]. This suggested that these phenotypes could be investigated with our proposed approach since it allows manipulating and tracking of approximately 2000 individual cells. In order to do so, we first loaded a 2-μL aliquot of a stationary phase *E. coli* culture into the microfluidic mother machine device [24] and confined the bacteria in the lateral channels of the device (*t* = 0, Fig. 1a, e). We then treated the bacteria with a high dose of ampicillin, 25× minimum inhibitory concentration (MIC), dissolved in Lysogeny broth (LB; 0 < *t* < 3 h, Fig. 1b). Since ampicillin targets cell wall synthesis, we identified susceptible lysed cells that die and lyse either during or after drug treatment and comprise the majority of the population (leftmost channel in Fig. 1) [28]. We then exchanged the drug-containing medium for LB (3 < *t* < 24 h, Fig. 1c) and identified intact persister cells that survived the antibiotic challenge and could regrow upon restoring favorable conditions (third channel from the left in Fig. 1), similarly to previously reported microfluidic approaches [6, 7, 18, 19]. However, these methodologies did not allow the study of VBNC cells. In order to overcome this limitation, we performed live/dead staining as a last step in the series of our on-chip assays [5, 29, 30]. This step allowed us to identify two further phenotypes of susceptible non-lysed cells that died but did not lyse during nor after drug treatment (rightmost channel in Fig. 1) and VBNC cells that survived the antibiotic challenge but did not regrow upon restoring favorable conditions (second channel from the left in Fig. 1). Since we imaged and tracked each single cell throughout this series of assays, we were able to measure the size and morphology of each single cell before, during and after drug treatment and to assign each cell to a specific phenotype at the end of the experiment. This is, to the best of our knowledge, the first methodology allowing for the study of VBNC cells, alongside persister and susceptible cells, before, during, and after drug treatment.

### Size and morphology of VBNC cells before, during, and after ampicillin treatment

**Fig. 2:**
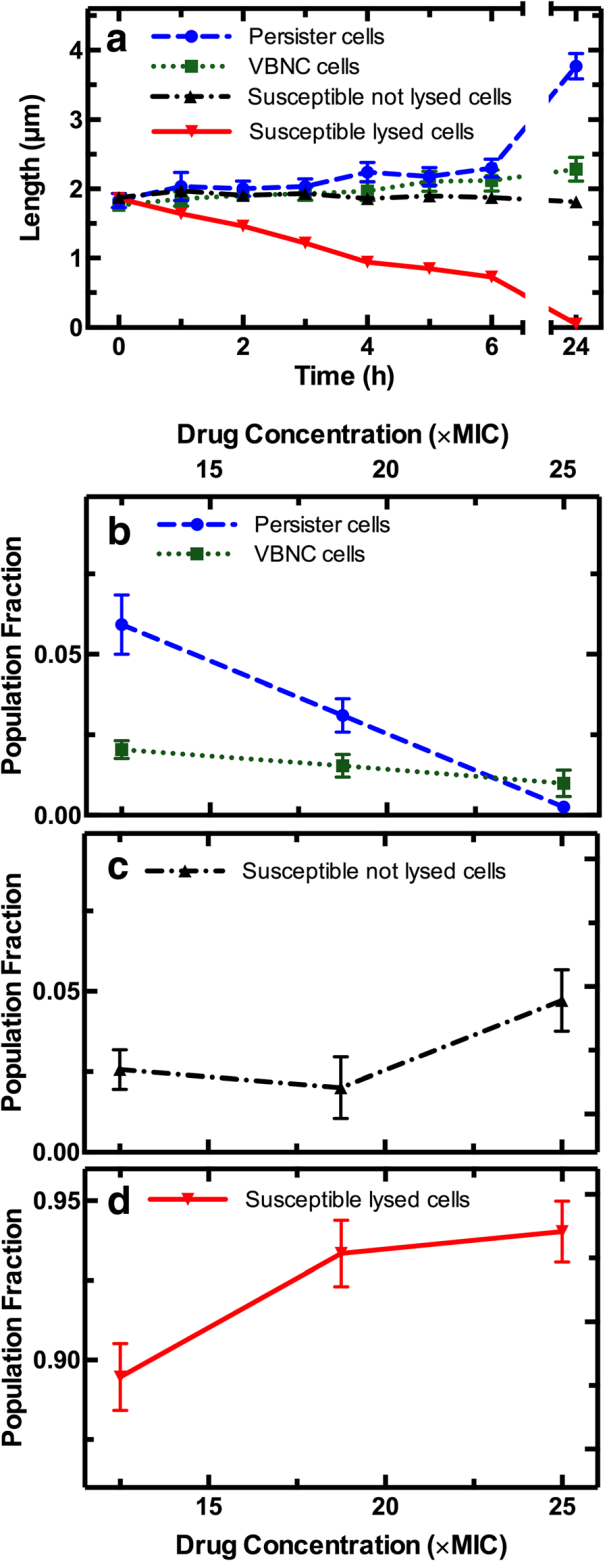
Dimension and fraction of viable but non-culturable (VBNC) cells. **a** Average length of VBNC cells (green squares), alongside persister (blue circles), susceptible non-lysed (black upward triangles), and susceptible lysed cells (red downward triangles) before (*t* = 0), during (0 < *t* < 3 h), and after treatment (3 < *t* < 24 h) with ampicillin at a concentration of 25 × minimum inhibitory concentration in Lysogeny broth. We assigned a length value of zero to cells that became undetectable with our code upon cell lysis. This explains the decrease in the average length of susceptible lysed cells. Lengths of untreated cells in control experiments are reported in Additional file 1: Figure S1a. Bacterial width did not significantly change throughout the experiment for the different phenotypes but for susceptible lysed cells (data not shown). Data and error bars are mean and standard error of the mean obtained by averaging single-cell values (*n*_*SL*_ = 1651, *n*_*P*_ = 33, *n*_*VBNC*_ = 48, *n*_*SNL*_ = 87) measured in the microfluidic experiment illustrated in Fig. 1 performed on biological triplicates (*N* = 3). We did not observe any significant difference between the results obtained from different biological replica. Due to the large sample sizes, error bars are small compared to the corresponding mean values and are hidden behind some of the data points. Ampicillin concentration dependence of the fraction of (**b**) VBNC and persister cells surviving the antibiotic challenge, of (**c**) susceptible non-lysed cells, and of (**d**) susceptible lysed cells killed by the antibiotic challenge. Data and error bars are obtained by averaging each phenotype frequency measured at least in biological triplicate

Before ampicillin treatment (*t* = 0), the four phenotypes (persister, VBNC, susceptible lysed, and susceptible non-lysed cells) had similar average lengths (Fig. 2a), calculated as the mean and standard error of the mean of single-cell measurements for the different phenotypes. Persister cells survived the 3-h ampicillin treatment and began dividing within 2 hours of exposure to LB (Additional file 1: Figure S1a); forming microcolonies and reaching an average length of 3.8 ± 0.2 μm by *t* = 24 h. In contrast, after exposure to ampicillin and subsequent incubation in LB, VBNC cells were smaller (2.3 ± 0.2 μm) in average length, (squares in Fig. 2a) and more rounded compared to persister cells. Indeed, both cell dwarfing and rounding have been observed in VBNC cells of many species [31, 32]. In separate control experiments we measured the growth of bacteria confined in the lateral channels of the mother machine device and exposed to LB, but without the addition of ampicillin (Additional file 1: Figure S1b, c). As expected, these bacteria exhibited similar initial average length to the bacteria measured in the ampicillin treatment experiments. However, they began dividing within 2 hours of exposure to LB (Additional file 1: Figure S1b) and at *t* = 24 h exhibited an average length close to that measured for the persister cell progenies (diamonds in Additional file 1: Figure S1c).

### VBNC cells constitute the majority of cells surviving ampicillin treatment in a stationary phase *E. coli* culture

We enumerated the bacteria belonging to the four phenotypes and defined *f*_*SL*_, *f*_*SNL*_, *f*_*P*_, and *f*_*VBNC*_ as the fractions given by the number of counts for susceptible lysed, susceptible non-lysed, persister, or VBNC cells, respectively, divided by the number of total cells imaged in our assay before drug treatment. When we used a high dose of ampicillin (25 × MIC), we measured *f*_*VBNC*_ = 0.01 and *f*_*P*_ = 0.003 (Fig. 2b). Therefore, we provide direct evidence that VBNC cells, rather than persister cells as recently reported [18], constitute the majority of cells surviving ampicillin treatment in a stationary phase *E. coli* culture [27]. These cells possibly entered the VBNC state during nutrient starvation in stationary phase [32] or during the successive antibiotic treatment [23]. These findings suggest the need for investigating the VBNC phenotype in concert with persister cells [4]. We also found that susceptible cells that did not lyse were another small fraction, while the majority of bacteria lysed as a result of drug treatment (*f*_*SNL*_ = 0.047 and *f*_*SL*_ = 0.94, Fig. 2c and d, respectively). We confirmed the reliability of our assay by performing bulk ampicillin treatment and colony forming unit (CFU) assays on separate aliquots of the same *E. coli* cultures. Through these bulk assays we obtained a measured *f*_*P*_ similar to that measured via our novel single-cell assay (Additional file 2: Figure S2) and to previously reported *f*_*P*_ values [33]. The CFU method does not, however, allow distinction between the VBNC and susceptible phenotypes. In contrast, our novel methodology provides a powerful tool to identify and study VBNC cells. Moreover, we found that *f*_*P*_ and *f*_*VBNC*_ decreased with ampicillin concentration (Fig. 2b and Additional file 2: Figure S2), whereas *f*_*SNL*_ and *f*_*SL*_ increased (Fig. 2c, d). It is worth noting that, at high ampicillin concentration (25 × MIC) we measured four times as many VBNC as persister cells, whereas at a lower ampicillin concentration (12.5 × MIC) this ratio was reversed, with *f*_*P*_ being three times higher than *f*_*VBNC*_ (Fig. 2b). These findings suggest the importance of employing the same standardized dose of drug when studying rare phenotypes within a clonal population.

### Tracking promoter activity in individual VBNC cells

In order to further investigate the VBNC state, we used the approach illustrated in Fig. 1 in combination with green fluorescent protein (GFP) transcriptional reporter strains [34]. Each strain carried a low copy number plasmid with the promoter region of a gene of interest inserted upstream of a gene for a fast-folding GFP with an average lifetime of 8 h. GFP expression levels in individual strains of this library have been used to measure the corresponding promoter activity both at the population and single cell levels [34, 35]. We used this approach to measure single-cell GFP fluorescence levels as a proxy for the activity of the promoters that initiate the transcription of the genes *tolC*, *tnaC*, and *ptsG*. The *tolC* gene encodes part of the multidrug efflux pump AcrAB-TolC, and *E. coli* persister cells have previously been shown to exhibit higher *tolC* expression than untreated cells [18]. The *tnaC* gene encodes the leader peptide of the *tnaCAB* operon responsible for tryptophan transport and catabolism, including the enzyme TnaA, which converts tryptophan to indole, pyruvate, and ammonia. Indole has previously been shown to regulate the formation of *E. coli* persister cells [9, 36]. The role of these putative persister genes in VBNC cells remains to be investigated. The *ptsG* gene encodes part of the glucose permease transporter, and was chosen as a control gene as it has not been previously associated with persister nor with VBNC cells.

**Fig. 3:**
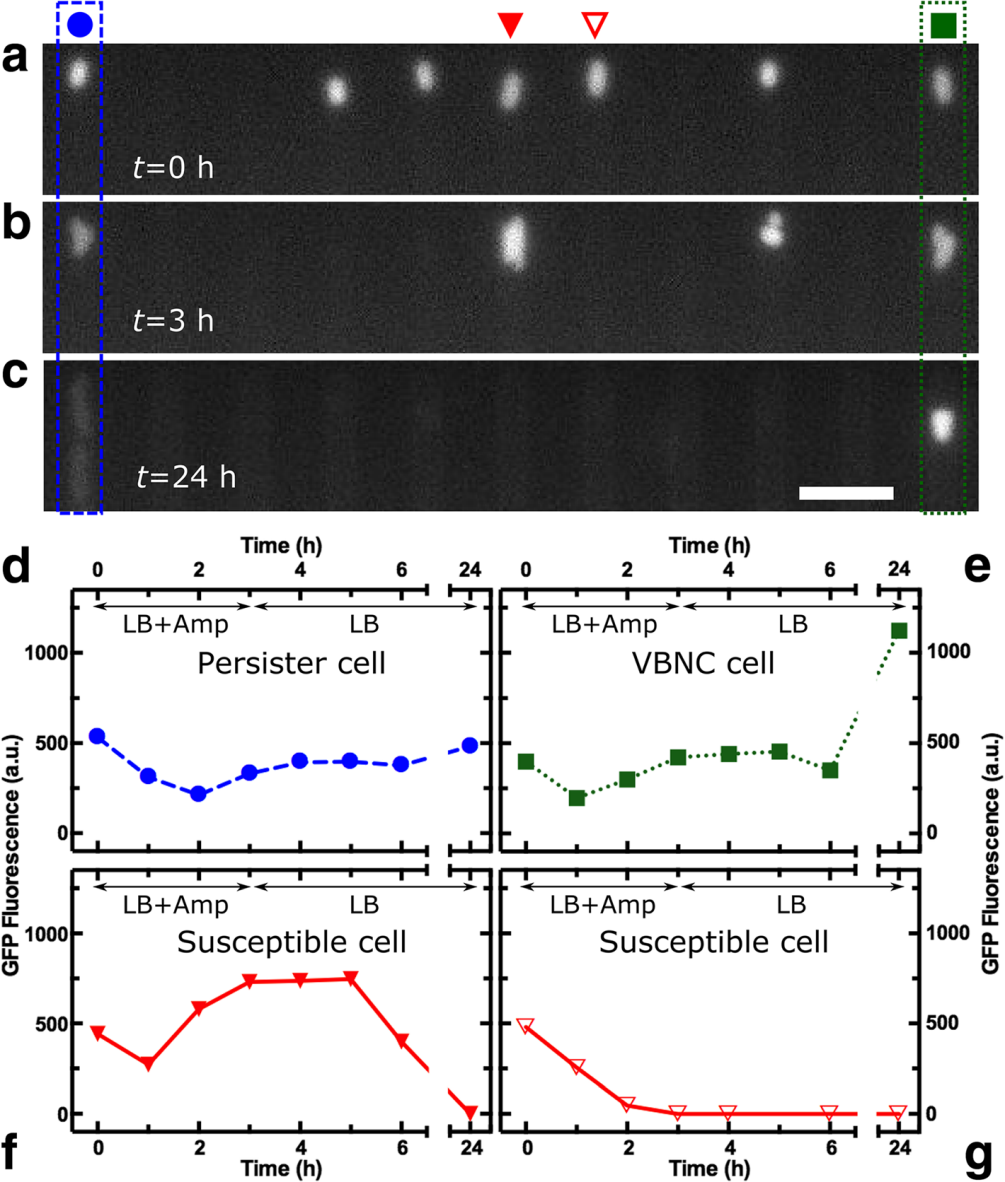
GFP fluorescence patterns in single viable but non-culturable (VBNC) cells. Fluorescence images reporting GFP expression levels, as a proxy for the activity of the promoter of *tolC*, in a single VBNC cell (green square), alongside a persister cell (blue circle) and five susceptible cells (**a**) before, (**b**) during, and (**c**) after drug treatment. Scale bar, 5 μm. Corresponding fluorescence patterns for the (**d**) persister, (**e**) VBNC, and (**f**, **g**) the two selected susceptible cells in the images above. The VBNC cell exhibited a fluorescence pattern similar to the one of the persister cell throughout the entire assay, but the VBNC cell was much brighter and easily distinguishable at *t* = 24 h. This suggests that the fluorescence associated to the *tolC* reporter strain could be used to isolate VBNC cells from the remainder of the population after ampicillin treatment and 21 h exposure to LB. The two susceptible cells displayed very different responses to the antibiotic treatment. For the cell in (f) (marked with a filled triangle in Fig. 3a), fluorescence levels increased during and immediately after drug treatment but decreased after *t* = 5 h, with the cell eventually lysing by *t* = 24 h. For the cell in (g) (marked with an open triangle in Fig. 3a), fluorescence levels decreased during drug treatment, with the cell lysing by *t* = 3 h. This suggests that besides the four phenotypes introduced in Figs. 1 and 2, there are at least two further subgroups of susceptible cells (Additional file 3: Figure S3)

Figure 3a–c shows a representative set of images displaying GFP fluorescence of a persister cell, a VBNC cell (dashed and dotted contours, respectively), and five susceptible lysed cells of the *tolC* reporter strain. Figure 3d–g shows the corresponding single-cell fluorescence throughout the different steps of the experiment, which are before (*t* = 0), during (0 < *t* < 3 h), and after (3 < *t* < 24 h) ampicillin treatment. We found that the VBNC cell exhibited a level of GFP fluorescence similar to the persister cell before and during drug treatment. After drug removal and 21 h exposure to LB, the VBNC cell was distinguishable from susceptible lysed or susceptible non-lysed cells even before performing the live/dead staining. In fact, whereas susceptible cells did not show any GFP fluorescence, the VBNC cell exhibited a high level of GFP fluorescence, being more than two times brighter than the persister cell progeny. This suggests that the non-growing VBNC phenotype maintains an active *tolC* promoter.

We also found that susceptible non-lysed and susceptible lysed cells showed similar patterns of fluorescence for the three investigated reporter strains and henceforth refer to them as susceptible cells. However, we identified two further subgroups within the susceptible subpopulation in the *tolC* reporter strain, namely cells that displayed increasing fluorescence levels during drug treatment (filled triangles in Fig. 3a–c, f and Additional file 3: Figure S3b) and cells that showed decreasing levels of fluorescence (open triangles in Fig. 3a–c, g and Additional file 3: Figure S3b).

### VBNC cells can be distinguished from susceptible cells before antibiotic treatment

In order to investigate the patterns of GFP fluorescence in VBNC cells of the three reporter strains, we imaged, tracked, and analyzed approximately 2000 individual bacteria for each reporter strain.

**Fig. 4:**
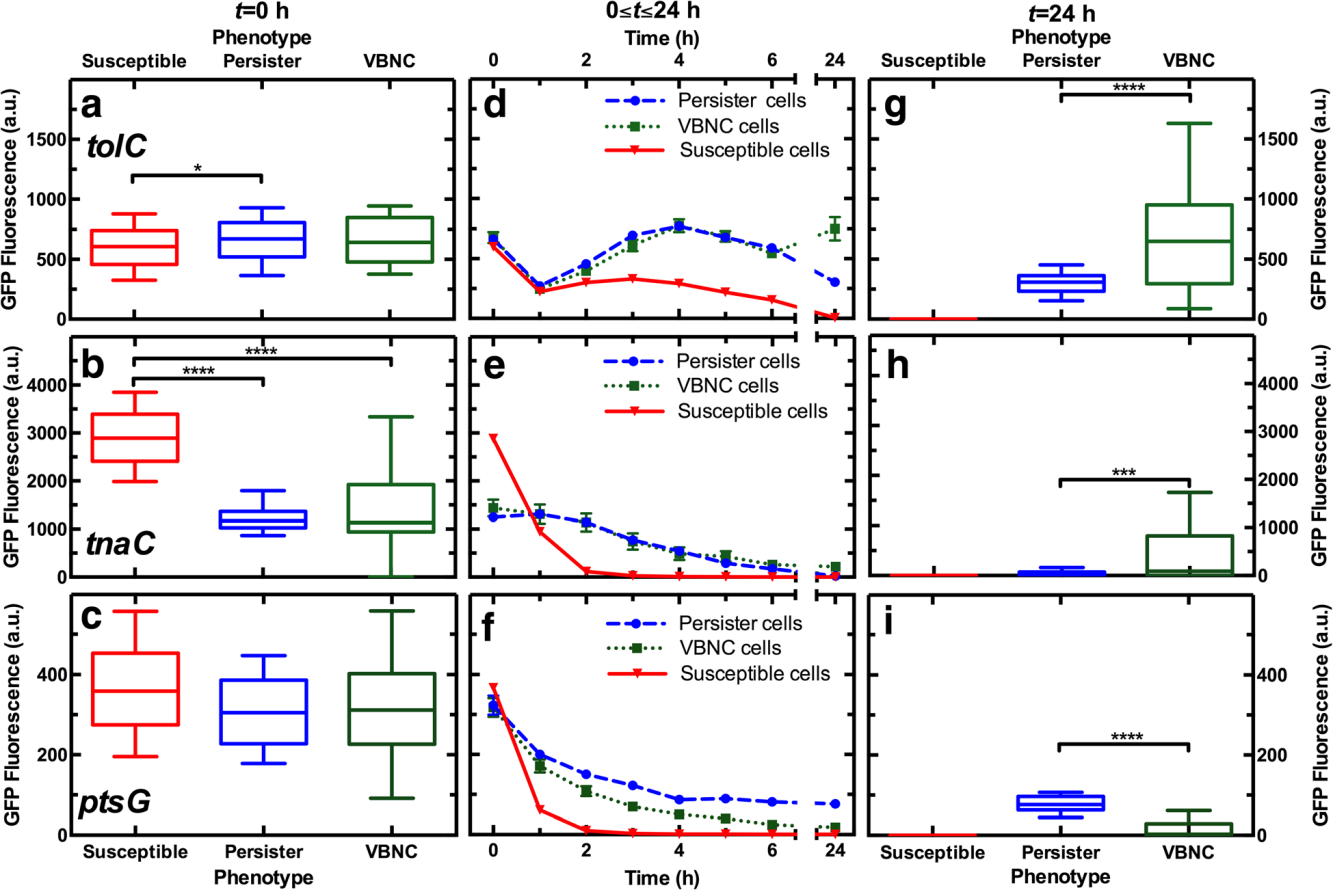
Distribution of GFP fluorescence levels in viable but non-culturable (VBNC) cells before and after drug treatment. Distribution of fluorescence levels in VBNC (green) cells, alongside the susceptible (red) and persister (blue) phenotypes in the (**a**) *tolC*, (**b**) *tnaC*, and (**c**) *ptsG* reporter strains before drug treatment (*t* = 0). Susceptible cells include both susceptible lysed and susceptible non-lysed phenotypes since these exhibit similar GFP fluorescence patterns. **d**–**f** Corresponding average pattern of fluorescence levels for the different phenotypes and reporter strains throughout the microfluidic assay, 0 < *t* < 24 h. **g**–**i** Distributions of fluorescence levels after drug removal and 21 h exposure to LB. The bottom and top of the box are the first and third quartiles, the band inside the box is the median, the bottom and top whiskers represent the 10th and 90th percentiles, respectively. Data are obtained at least in biological triplicate (*N* = 3) for each reporter strain employed for a total of *n*_*S*_ = 6659, *n*_*P*_ = 198, and *n*_*VBNC*_ = 147 fluorescence patterns of single susceptible, persister, and VBNC cells, respectively. We did not observe any significant difference between the results obtained from different biological replica. Due to the large sample sizes, error bars are small compared to the corresponding mean values and are hidden behind some of the data points in (**d**), (**e**) and (**f**). The differential *tnaC* fluorescence levels in VBNC cells with respect to susceptible cells allows identifying the former as the dimmest cells before drug treatment, establishing *tnaC*-GFP as a potential biomarker to isolate VBNC cells, alongside persister cells, within the untreated clonal population. Moreover, at *t* = 24 h, VBNC cells display increased fluorescence levels in the *tolC* and *tnaC* reporter strains, whereas persister cells show increased fluorescence levels in the *ptsG* reporter strain. Therefore, the three investigated reporter strains could be used in future as biomarkers to distinguish and isolate VBNC from persister cells after drug challenge and exposure to LB, these two phenotypes often being confused

Before ampicillin treatment, VBNC cells exhibited fluorescence levels similar to those measured in persister cells for all the investigated reporter strains (Fig. 4a–c). This further supports the hypothesis that the persister and VBNC phenotypes are part of a shared physiological continuum [4]. Furthermore, persister and VBNC cells of the *tolC* reporter strain exhibited only slightly higher fluorescence levels than susceptible cells (Fig. 4a). This suggests that persister and VBNC cells may not rely on antibiotic efflux to survive the antibiotic challenge in disagreement with previously reported findings [18]. This disagreement, however, could also be ascribed to the use of different antibiotic treatment conditions. Remarkably, fluorescence levels in persister and VBNC cells in the *tnaC* reporter strain were half of those measured in susceptible cells, possibly because the former are in a less active metabolic state [27]. This contradicts a previous study that suggested that persister cells exhibit higher levels of *tnaC* promoter activity with respect to susceptible cells [9]; however, in that study, 0.5 mM indole had been added to an exponentially growing *E. coli* culture. Furthermore, a different study suggested a link between reduction of *tnaCAB* operon expression and the formation of persister cells [36], corroborating our findings. Our data suggests that the persister and VBNC cells found in a stationary phase *E. coli* culture constitute subpopulations existing before exposure to ampicillin, confirming previous findings [6]. However, this is still under debate since other studies suggest that exposure to antibiotics itself underlies persister cell formation [12]. We anticipate that our new methodology will be crucial for identifying further phenotypic differences between VBNC, persister, and susceptible cells prior to drug treatment.

During ampicillin treatment, VBNC and persister cells maintained similar fluorescence levels in the *tolC* and *tnaC* reporter strains, but VBNC cells displayed lower fluorescence levels in the *ptsG* reporter strain compared to persister cells (circles and squares in Fig. 4d–f, 0 < *t* < 3 h). On the contrary, susceptible cells very quickly lost their fluorescence in all three employed reporter strains (triangles in Fig. 4d–f) but not in a subpopulation of the *tolC* reporter strain (Fig. 4d and Additional file 3: Figure S3).

After ampicillin treatment and 21 h exposure to LB, VBNC cells displayed markedly different fluorescence levels for all the investigated strains compared to persister cells. VBNC cells maintained higher fluorescence levels in the *tolC* and *tnaC* reporter strains, and exhibited different fluorescence levels with respect to susceptible cells (Additional file 4: Figure S4). This is consistent with the hypothesis that, whereas dead cells do not express genes, VBNC cells continue producing mRNA [37]. Therefore, we unambiguously demonstrated that VBNC cells can be distinguished from susceptible non-lysed cells before, during, and after drug treatment. Persister cells instead showed higher fluorescence levels in the *ptsG* reporter strain (Fig. 4g–i) compared to VBNC cells, suggesting that the former were utilizing a higher amount of sugars after drug treatment.

In separate control experiments we measured the changes in GFP fluorescence of bacteria confined in the lateral channels of the mother machine device, exposing them to LB without the addition of ampicillin. As expected, these bacteria exhibited similar fluorescence levels to susceptible cells at *t* = 0 (Additional file 5: Figure S5). Moreover, during exposure to LB, these bacteria exhibited similar patterns of fluorescence levels as persister cells (Additional file 6: Figure S6, 3 < *t* < 24 h), further suggesting that persister cells had reverted to a normally growing state.

## Discussion

Progress in our understanding of VBNC cells since their discovery in 1982 [38] has been slow and incremental. Our approach represents a general tool to measure the physiological state of VBNC cells, alongside persister cells, before antibiotic treatment. Moreover, this approach, as well as other recently introduced methods [27], can serve to bridge the gap between VBNC and persister states that are rarely studied in the same context [4]. Specifically, in this paper we quantitatively compare persister and VBNC fractions in a stationary phase *E. coli* culture, and characterize the size, morphology, and activity of selected promoters in persister and VBNC cells exposed to the same microenvironment. However, our approach could easily be adapted to measure other physiological parameters previously linked to the formation of VBNC and persister cells such as uptake and efflux of nutrients and drugs [18, 39], metabolic activity [5], and mRNA metabolism [40].

Little is known about the genetic control underlying the VBNC state and the gene expression profile in VBNC *E. coli* [3, 41, 42]. Indeed, transcriptome analysis has been reported only for *V. cholerae* VBNC cells [43]. Furthermore, gene expression profiling is typically carried out on VBNC cells isolated after exposure to drugs [27] or other stressful environments such as cold seawater or low pH [3, 43], which affect the VBNC state [44]. Our approach instead allows investigating VBNC cells, alongside persister cells, before drug treatment and in a microenvironment that we can accurately control via microfluidics. In this respect, our approach is likely to change the way we investigate non-growing subpopulations within clonal or mixed microbial populations.

It is worth noting that, if VBNC cells are present in microbiological quality control samples from the food industry, water distribution systems, or hospitals, the number of viable bacteria in the sample will be underestimated via the widely employed CFU count method. Therefore, the inability to detect VBNC cells can pose a risk to human health [3]. Our platform represents a rapid and reliable tool to detect VBNC cells in bacterial populations. This allowed us to unambiguously demonstrate that VBNC cells are not in a transitory stage in the degeneration of bacteria leading to cell death [45, 46]. On the contrary, our data proves that, even after drug treatment, at least the *tolC* promoter remained active in VBNC cells and thus supports the hypothesis that VBNC cells are in a transitional state awaiting for suitable conditions to resuscitate [44]. In this respect, our single-cell approach represents an ideal tool for the future investigation of the mechanisms underlying VBNC resuscitation, which remain largely unknown [3, 47].

Our microfluidic system can be adapted to investigate other bacterial pathogens and cell types, including cancer populations where drug-tolerant cells have been identified [48]. More generally, our platform will allow the investigation of how non-growing subpopulations within clonal or mixed microbial populations respond to changes in the environmental pH, temperature, and nutrient content. Furthermore, we are currently working on scaling up our microfluidic system to investigate persister and VBNC phenotypes occurring at frequencies below 10^-3^. Specifically, we plan to apply our approach to bacterial cultures transitioning from the exponential to the stationary phase when the fraction of persister, and possibly VBNC cells, monotonously increases from 10^-5^ to 10^-2^ [26], thus suggesting that the changes occurring in the culture environment might favor the formation of these phenotypes. Such a higher throughput system will also allow investigating candidate VBNC cell formation mechanisms involving multiple genes through the simultaneous investigation of several reporter strains [4]. In this respect, a recent study investigated the expression of up to 100 genes in persister cells, but not in VBNC cells, by using fluorescence-activated cell sorting, sequencing, and a library of promoter reporters [33]. However, this approach also relies on the use of antibiotics to identify persister cells and does not allow measurements of gene expression on the same cell before, during, and after drug treatment.

## Conclusion

There is currently a lack of biomarkers that can be used to segregate VBNC cells from the majority of S cells before antibiotic treatment [33]. Our single-cell approach allowed us to establish that, when using the *tnaC* reporter strain, stationary phase persister and VBNC *E. coli* were significantly less fluorescent than susceptible *E. coli* and will allow future identification of other genes that are differentially regulated in VBNC cells before antibiotic treatment. The associated reporter strains could be used to isolate natively formed VBNC cells via fluorescence-activated cell sorting for downstream transcriptomic and proteomic analysis. As such, our novel single-cell approach provides a new opportunity to unravel the molecular mechanisms underlying the capability of microbial pathogens to survive drug treatment and remain non-growing.

## Methods

### Chemicals and cell culture

All chemicals were purchased from Fisher Scientific or Sigma-Aldrich unless otherwise stated. LB medium (10 g/L tryptone, 5 g/L yeast extract, and 10 g/L NaCl) and LB agar plates (LB with 15 g/L agar) were used for planktonic growth and enumeration of CFUs, respectively. *E. coli* BW25113 were purchased from Dharmacon (GE Healthcare). *tolC*, *tnaC*, and *ptsG* reporter strains of an *E. coli* K12 MG1655 promoter library [34] were purchased from Dharmacon (GE Healthcare). Plasmids were extracted and transformed into chemically competent *E. coli* BW25113 as previously reported [33]. We verified that these reporter strains exhibit similar growth rates and levels of *f*_*P*_ and *f*_*VBNC*_ compared to wild-type *E. coli* BW25113. Overnight cultures were prepared by picking a single colony of *E. coli* BW25113 from a streak plate and growing it in 200 mL fresh LB medium in a shaking incubator at 200 rpm and 37 °C for 17 h.

### Fabrication of the microfluidic devices

The mold for the mother machine microfluidic device [24] was obtained by pouring epoxy onto a polydimethylsiloxane (PDMS, Dow Corning) replica of the original mold containing 12 independent microfluidic chips (kindly provided by S. Jun). Each of these chips is equipped with approximately 6000 lateral microfluidic channels with width and height of approximately 1 μm each and a length of 25 μm. These lateral channels are connected to a main microfluidic chamber that is 25 and 100 μm in height and width, respectively. PDMS replicas of this device were realized as previously described [49]. Briefly, a 9:1 (base:curing agent) PDMS mixture was cast on the mold and cured at 70 °C for 120 min in an oven. The cured PDMS was peeled from the epoxy mold and cut into individual chips. Fluidic accesses were created by using a 0.75 mm biopsy punch (Harris Uni-Core, WPI). The PDMS chip was irreversibly sealed on a glass coverslip by exposing both surfaces to oxygen plasma treatment (10 s exposure to 30 W plasma power, Plasma etcher, Diener, Royal Oak, MI, USA). This treatment temporarily rendered the PDMS and glass hydrophilic, so within 5 min after bonding the chip was filled with 2 μL of a 50 mg/mL bovine serum albumin solution and incubated at 37 °C for 1 h, thus passivating the device’s internal surfaces and preventing subsequent cell adhesion.

### Microfluidics-microscopy assay to identify and study VBNC cells

An overnight culture was prepared as described above. Spent LB broth and bacteria were prepared by centrifuging the overnight culture (10 min at 3000 *g* and 20 °C). The supernatant was filtered twice (Medical Millex-GS Filter, 0.22 μm, Millipore Corp.) and used to re-suspend the bacteria in their spent LB to an optical density of 50 at 595 nm. Bovine serum albumin was added to the bacterial suspension at a concentration of 0.5 mg/mL. A 2-μL aliquot of this suspension was injected in the above described microfluidic device and incubated at 37 °C. The high bacterial concentration favors bacteria entering the narrow lateral channels from the main microchamber of the mother machine (Fig. 1a, e) [24]. We found that an incubation time of 20 min allowed filling of the lateral channels with, typically, between one and three bacteria per channel. An average of 60% of lateral channels of the mother machine device were filled with bacteria and 50% of the filled channels contained single bacteria. This facilitated tracking each single bacterium throughout the different phases of our assay (Fig. 1). The microfluidic device was completed by the integration of fluorinated ethylene propylene tubing (1/32" × 0.008"). The inlet tubing was connected to a flow-rate measuring device (Flow Unit S, Fluigent, Paris, France) controlling the pressure applied by a computerized pressure-based flow control system (MFCS-4C, Fluigent) on the inlet reservoir feeding the flow rate device itself. This instrumentation was controlled by MAESFLO software (Fluigent). At the end of the 20 min incubation period, the chip was mounted on an inverted microscope (IX73 Olympus, Tokyo, Japan) and the bacteria remaining in the main microchamber of the mother machine were washed into the outlet tubing and reservoir by flowing spent LB at 300 μL/h for 8 minutes. At this point (Fig. 1a, e) we acquired our first set of images in bright-field and fluorescence mode (the latter were acquired only when using reporter strains). Images were collected via a 60×, 1.2 NA objective (UPLSAPO60XW, Olympus) and a sCMOS camera (Zyla 4.2, Andor, Belfast, UK). The region of interest of the camera was adjusted to visualize 23 lateral channels per image. Upon acquiring each bright-field image the microscope was switched to fluorescent mode and FITC filter using custom built Labview software and a fluorescence image was acquired by exposing the bacteria to the blue excitation band of a broad-spectrum LED (CoolLED pE300white, Andover, UK) at 100%, 20%, and 20% of its intensity and for 0.05, 0.03, and 0.05 s for *tolC*, *tnaC*, and *ptsG* imaging, respectively. These parameters were adjusted in order to maximize the signal to noise ratio. The device was moved by two automated stages (M-545.USC and P-545.3C7, Physik Instrumente, Karlsruhe, Germany, for coarse and fine movements, respectively) to image the next set of lateral channels and these steps were repeated until approximately 2000 bacteria were imaged (average of 1943 ± 316 bacteria per experiment). After acquiring the first set of images, the microfluidic environment was changed by flowing LB containing ampicillin at 25× MIC at 300 μL/h for 8 minutes and then at 100 μL/h for 3 h (*t* = 0, Fig. 1b). Imaging was carried out hourly for 0 < *t* < 6 h and at *t* = 24 h. After 3 h, the antibiotic was removed and LB medium was flowed in the chip at 300 μL/h for 8 minutes and then at 100 μL/h for 21 h (Fig. 1c). Crucially, at *t* = 24 h live/dead staining [27] was performed by flowing SYTO9 and propidium iodide (PI, Thermo Fisher Scientific) into the microfluidic device for 25 and 10 min, respectively, at a concentration of 3.34 and 30 μM, respectively, according to the manufacturer’s specifications. To the best of our knowledge, this is the first time that live/dead staining is implemented in the mother machine approach and it is crucial for distinguishing VBNC cells from susceptible non-lysed cells. Indeed, these fluorescent dyes differ in their ability to penetrate the bacterial membrane and stain live and dead bacterial cells, respectively [50]. Bright-field and fluorescence images were acquired to determine whether each bacterium was dead or alive. SYTO9 and PI staining were imaged by using 60% blue and 100% green LED intensity, FITC and TRITC filters, respectively, and an exposure time of 0.01 s. PI only was used imaging reporter strains. The entire assay was carried out at room temperature.

### Image and data analysis

Microfluidics confinement and time-lapse microscopy allow the tracking of each individual bacterium and its eventual progeny throughout the entire assay. All image processing was performed using a custom Python module [51]. Briefly, channels and bacteria were detected by using automated thresholding algorithms. Each channel within a frame and each bacterium within a channel were assigned unique numeric labels. Each bacterium was tracked by taking into account all of the possible combinations for cell death or division and the most likely calculated depending on several factors, including each cell location and area. Once bacterial lineage was established, the corresponding masks were used to extract information about each bacterium such as width, length, area and fluorescence intensity from the corresponding fluorescence image. The fluorescence background was determined as the average fluorescence for the areas of the channels that did not contain bacteria and subtracted from the fluorescence intensity measured on each bacterium. For each bacterium, the measured GFP signal was normalized by cell size to account for variations in expression due to the cell cycle [52]. The final output images showed each detected bacterium with its unique numeric label. The measurements for all bacteria within a frame across the different time points were written to .csv files. The detection and label assignment was visually verified and each bacterium classified as persister, VBNC, susceptible lysed, or susceptible non-lysed phenotype as defined in Fig. 1. Further details of this framework will be reported in a separate publication currently in preparation. Semi-automated measurements were performed on randomly selected pools of images using ImageJ and the accuracy of the above automated framework verified. All data reported in GraphPad Prism 7 represent mean and standard error of the mean of at least biological triplicates. Due to the large sample sizes, error bars are small compared to the corresponding mean values and are hidden behind the data points in some of the graphs (Fig. 4d–f, Additional file 1: Figure S1a, Additional file 3: Figure S3b, and Additional file 4: Figure S4). Statistical significance was tested by unpaired *t* test with Welch’s correction.

### MIC determination and persister enumeration via CFU assay

The MIC of ampicillin against *E. coli* strain BW25113 was determined using a 96-well plate method. *E. coli* was grown in LB medium for 17 h at varying concentrations of ampicillin (0.5–512 μg mL^-1^) and the absorbance measured at 595 nm. The MIC was defined as the lowest concentration at which the absorbance was the same as the control (bacteria-free LB) and determined as 5 μg mL^-1^. In order to measure the fraction of persister cells to ampicillin in the culture, six 500-μL aliquots were withdrawn from the overnight culture. Three were used for untreated controls, centrifuged (12,000 *g* for 5 minutes), the supernatant removed, the pellet re-suspended in phosphate-buffered saline, serially diluted, plated on LB agar, and CFU counted after overnight growth in an incubator at 30 °C. Three aliquots were supplied with 500 μL LB containing ampicillin at 50 × MIC (resulting in a final concentration of 25 × MIC) and returned to the shaking incubator. After 3 h, these aliquots were centrifuged, the supernatant removed, the pellet re-suspended in phosphate-buffered saline, serially diluted, plated on LB agar, and CFU counted after overnight growth in an incubator at 30 °C. The persister cell fraction *f*_*P*_ was defined as the number of CFUs obtained from the treated aliquots divided by the number of CFUs obtained from the untreated aliquots.

## Additional files

Additional file 1: Figure S1. Bacterial growth in the microfluidic device. a) Fractional distributions of time elapsed to first cell division for persister cells. b) Fractional distributions of time elapsed to first cell division for untreated cells in separate control experiments where LB but without ampicillin was added in the mother machine device. *n* ≥ 3, *n*_*P*_ = 134 and *n*_*C*_ = 1274 persister and untreated control cells, respectively. c) Corresponding average length of persister cells (circles) before (*t* = 0), during (0 < *t* < 3 h), and after treatment (3 < *t* < 24 h) with ampicillin at a concentration of 25 × MIC in LB and of untreated control cells (diamonds). Data and error bars are mean and standard error of the mean obtained by averaging single-cell values obtained on *n*_*P*_ = 33 and *n*_*C*_ = 592 persister and untreated control cells, respectively, in biological triplicate. We did not observe any significant differences between the results obtained from different biological replica. Due to the large sample sizes, error bars are small compared to the corresponding mean values and are hidden behind some of the data points in (c). (PNG 1509 kb)

Additional file 2: Figure S2. Bulk and single-cell persister enumeration. Dependence of the frequency of persister cells on ampicillin concentration as measured via the single-cell microfluidics-microscopy assay (full circles) illustrated in Fig. 1 and the colony forming unit assay (open circles). The two assays are performed on aliquots withdrawn from the same *E. coli* overnight culture. Data and error bars are mean and standard error of the mean of measurements obtained in biological triplicate (*N* = 3). Data agreement within experimental error confirms the validity of the newly developed microfluidic assay. (PNG 226 kb)

Additional file 3: Figure S3. Subgroups in the subpopulation of susceptible cells. a) Distribution of GFP fluorescence levels in susceptible cells of the *tolC* reporter strain after ampicillin treatment (*t* = 3 h). b) Temporal dependence of average fluorescence levels for susceptible cells that fluoresce (filled triangles) and do not fluoresce (open triangles) at *t* = 3 h, resembling the two susceptible cells reported in Fig. 3. Data and error bars are the mean and standard error of *n* = 623 (filled triangles) and *n* = 754 (open triangles) susceptible cells measured in biological triplicate (*N* = 3). We did not observe any significant difference between the results obtained from different biological replica. Due to the large sample sizes, error bars are small compared to the corresponding mean values and are hidden behind the data points in (b). (PNG 365 kb)

Additional file 4: Figure S4. Viable but non-culturable (VBNC) cells are different from susceptible non-lysed cells (SNL). Average pattern of GFP fluorescence levels of the VBNC (green squares) and SNL (black triangles) phenotypes throughout the microfluidic assay for the a) *tolC*, b) *tnaC*, and c) *ptsG* reporter strains. The two phenotypes are already distinguishable during drug treatment. Data and error bars are obtained as the mean and standard error of the mean of single-cell measurements in biological triplicate (*N* = 3) for each reporter strain for a total of *n*_*VBNC*_ = 147 and *n*_*SNL*_ = 335 VBNC and SNL cells, respectively. We did not observe any significant difference between the results obtained from different biological replica. Due to the large sample sizes, error bars are small compared to the corresponding mean values and are hidden behind some of the data points. (PNG 568 kb)

Additional file 5: Figure S5. Susceptible cells are indistinguishable from untreated cells before drug treatment. Distribution of fluorescence levels in the susceptible phenotype before drug treatment (*t* = 0) and in untreated control cells before regrowth in LB (*t* = 0) in the a) *tolC*, b) *tnaC*, and c) *ptsG* reporter strain. The two populations are not statistically different, an unpaired *t* test with Welch’s correction yielding a *P* value of 0.07, 0.7, and 0.9, respectively. The bottom and top of the box are the first and third quartiles, the band inside the box is the median, the bottom and top whiskers represent the 10th and 90th percentiles, respectively. Data are obtained at least in biological triplicate (*N* = 3) for each reporter strain employed for a total of *n*_*S*_ = 6659 and *n*_*C*_ = 3076 susceptible and control cells, respectively. We did not observe any significant difference between the results obtained from different biological replica. (PNG 423 kb)

Additional file 6: Figure S6. Persister and untreated control cells have similar patterns of fluorescence levels during regrowth on LB. Average pattern of fluorescence levels in persister (circles) and untreated control (diamonds) cells throughout the microfluidic assay in the a) *tolC*, b) *tnaC*, and c) *ptsG* reporter strains. Persister and untreated control cells exhibit similar patterns of fluorescence levels during regrowth on LB (3 < *t* < 24 h and 0 < *t* < 24 h for persister and control cells, respectively) demonstrating that persister cells revert back to a normally growing state after removal of the antibiotic drug. Data and error bars are obtained as the mean and standard error of the mean of single-cell measurements in biological triplicate (*N* = 3) for each reporter strain for a total of *n*_*P*_ = 132 and *n*_*C*_ = 3076 persister and control cells, respectively. We did not observe any significant difference between the results obtained from different biological replica. Due to the large sample sizes, error bars are small compared to the corresponding mean values and are hidden behind the data points. (PNG 585 kb)
