# Supplementary figures and images for: Investigating the physiology of viable but non-culturable bacteria by microfluidics and time-lapse microscopy
Source: BMC Biol. 2017 Dec 21;15:121. doi: 10.1186/s12915-017-0465-4 (PMC5738893; doi:10.1186/s12915-017-0465-4)

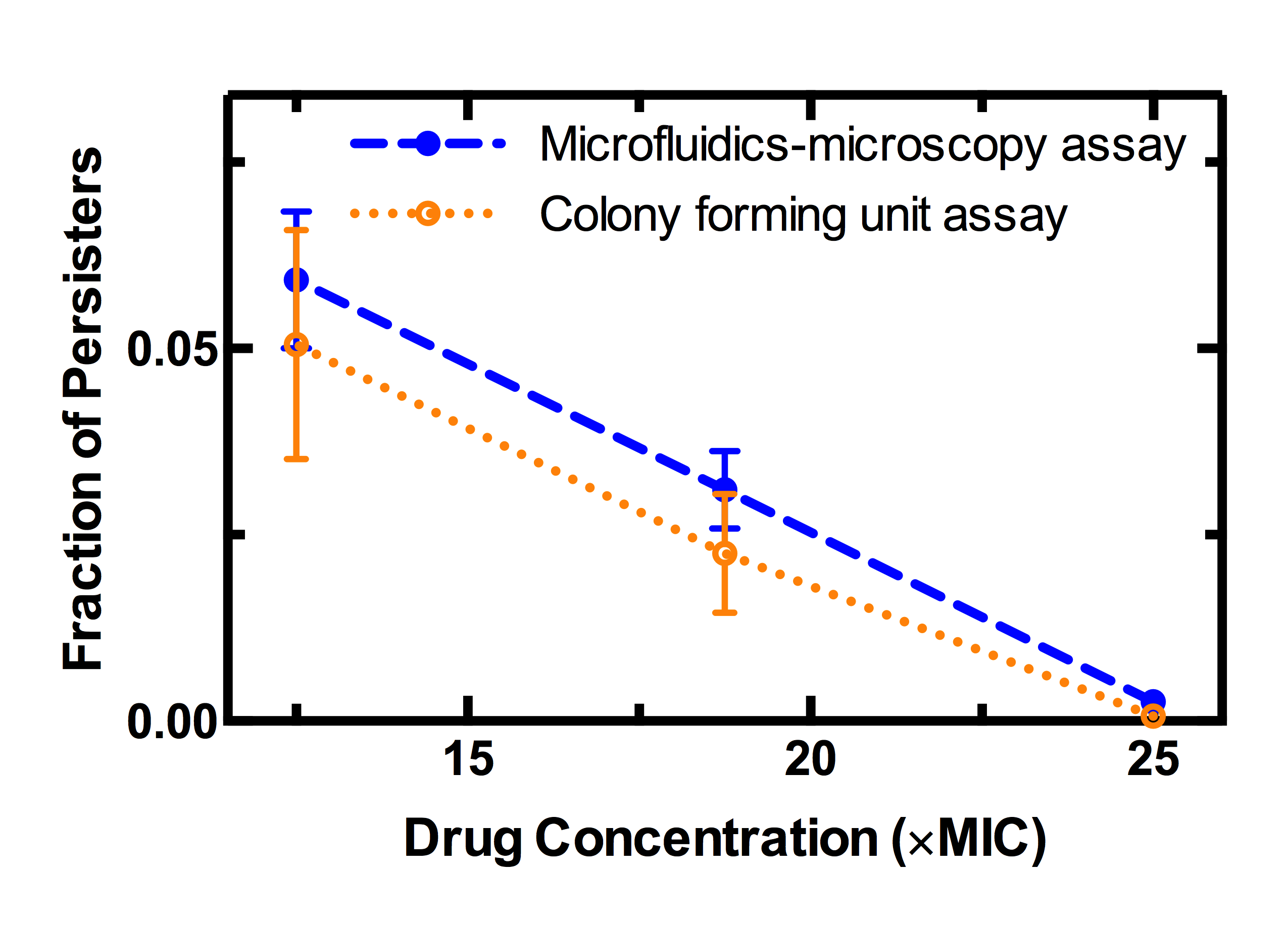

Supplement: Supplementary file 2 — Bulk and single-cell persister enumeration. Dependence of the frequency of persister cells on ampicillin concentration as measured via the single-cell microfluidics-microscopy assay (full circles) illustrated in Fig. 1 and the colony forming unit assay (open circles). The two assays are performed on aliquots withdrawn from the same E. coli overnight culture. Data and error bars are mean and standard error of the mean of measurements obtained in biological triplicate (N = 3). Data agreement within experimental error confirms the validity of the newly developed microfluidic assay. (PNG 226 kb) [file 12915_2017_465_MOESM2_ESM.png]

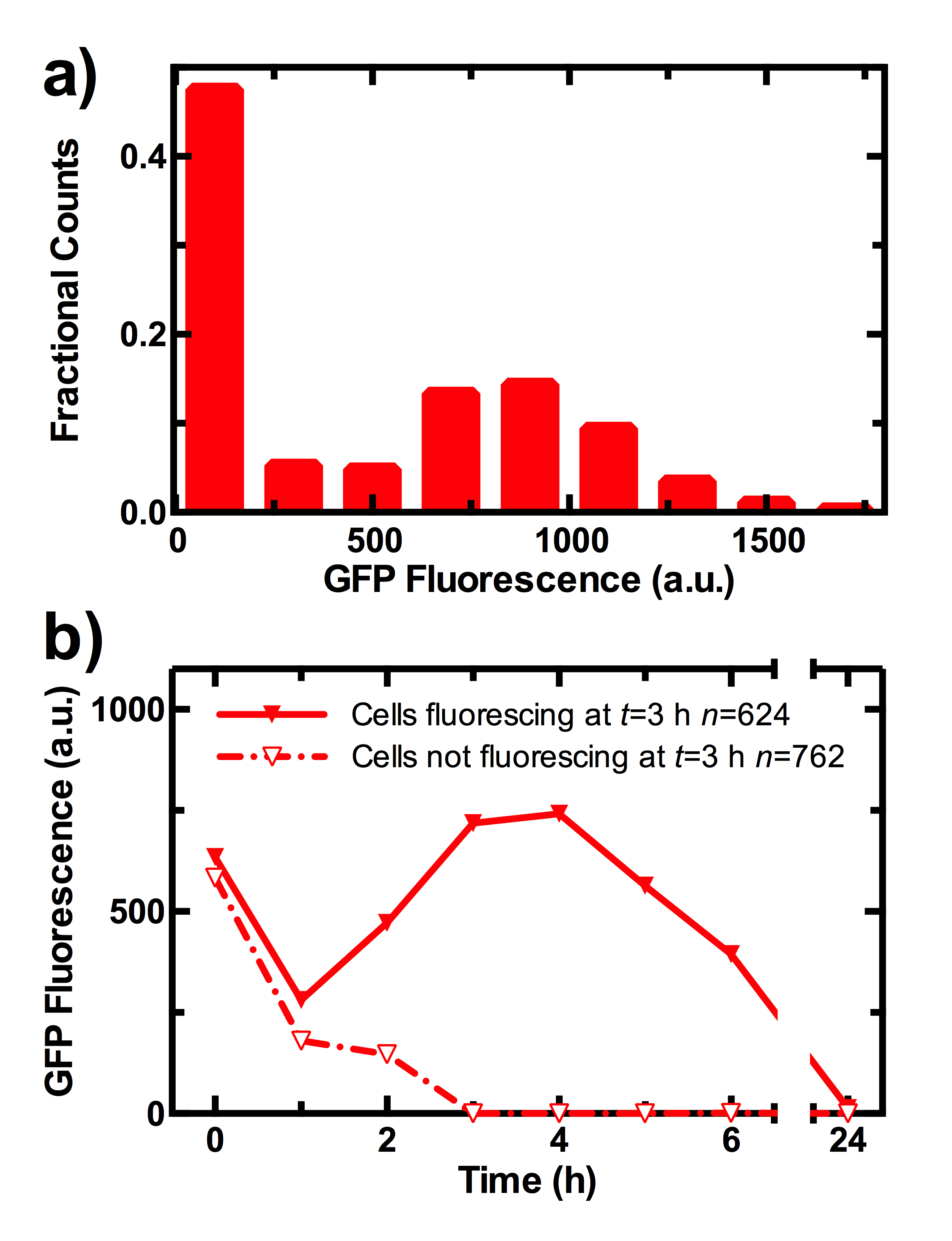

Supplement: Supplementary file 3 — Subgroups in the subpopulation of susceptible cells. a) Distribution of GFP fluorescence levels in susceptible cells of the tolC reporter strain after ampicillin treatment (t = 3 h). b) Temporal dependence of average fluorescence levels for susceptible cells that fluoresce (filled triangles) and do not fluoresce (open triangles) at t = 3 h, resembling the two susceptible cells reported in Fig. 3. Data and error bars are the mean and standard error of n = 623 (filled triangles) and n = 754 (open triangles) susceptible cells measured in biological triplicate (N = 3). We did not observe any significant difference between the results obtained from different biological replica. Due to the large sample sizes, error bars are small compared to the corresponding mean values and are hidden behind the data points in (b). (PNG 365 kb) [file 12915_2017_465_MOESM3_ESM.png]

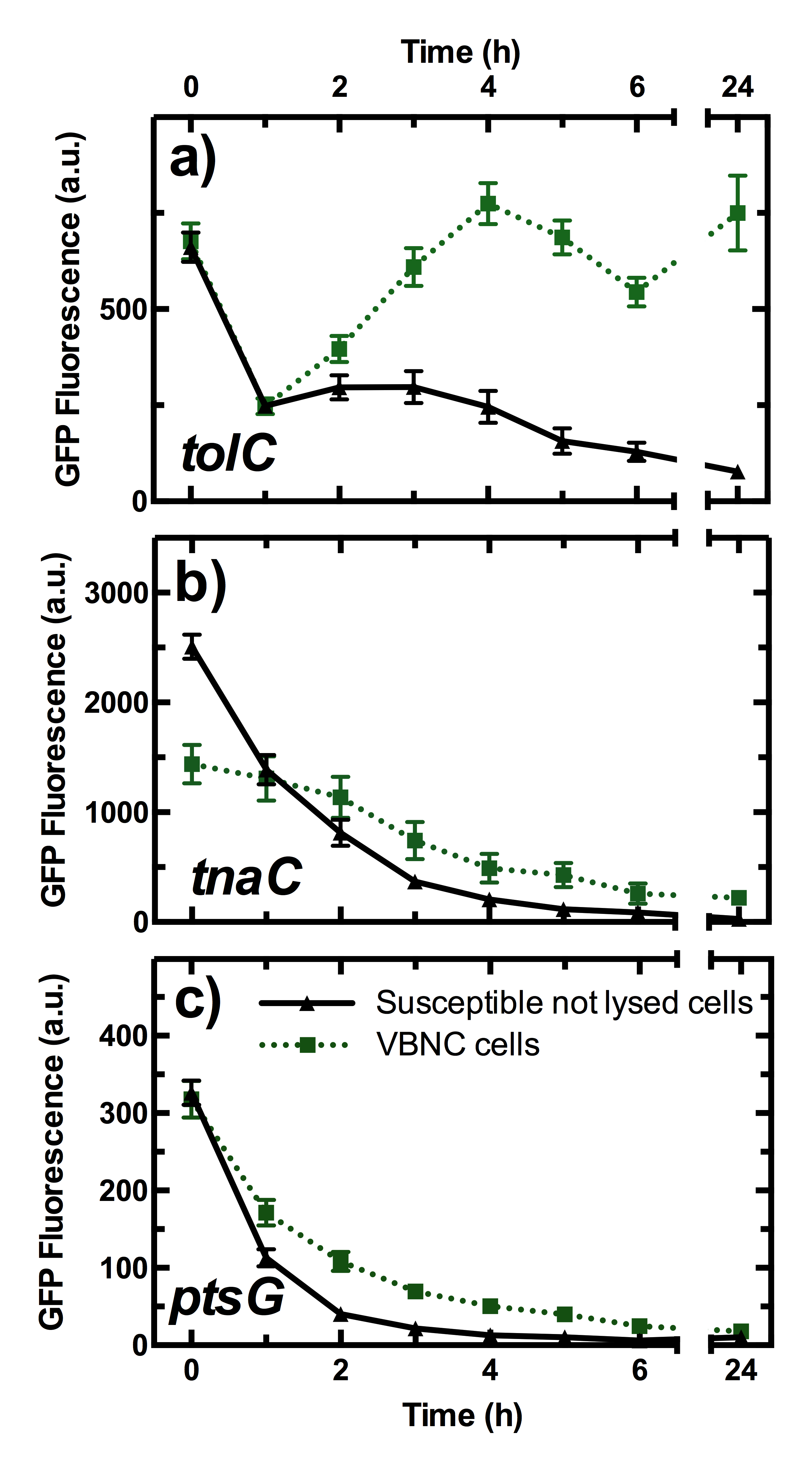

Supplement: Supplementary file 4 — Viable but non-culturable (VBNC) cells are different from susceptible non-lysed cells (SNL). Average pattern of GFP fluorescence levels of the VBNC (green squares) and SNL (black triangles) phenotypes throughout the microfluidic assay for the a) tolC, b) tnaC, and c) ptsG reporter strains. The two phenotypes are already distinguishable during drug treatment. Data and error bars are obtained as the mean and standard error of the mean of single-cell measurements in biological triplicate (N = 3) for each reporter strain for a total of n VBNC = 147 and n SNL = 335 VBNC and SNL cells, respectively. We did not observe any significant difference between the results obtained from different biological replica. Due to the large sample sizes, error bars are small compared to the corresponding mean values and are hidden behind some of the data points. (PNG 568 kb) [file 12915_2017_465_MOESM4_ESM.png]

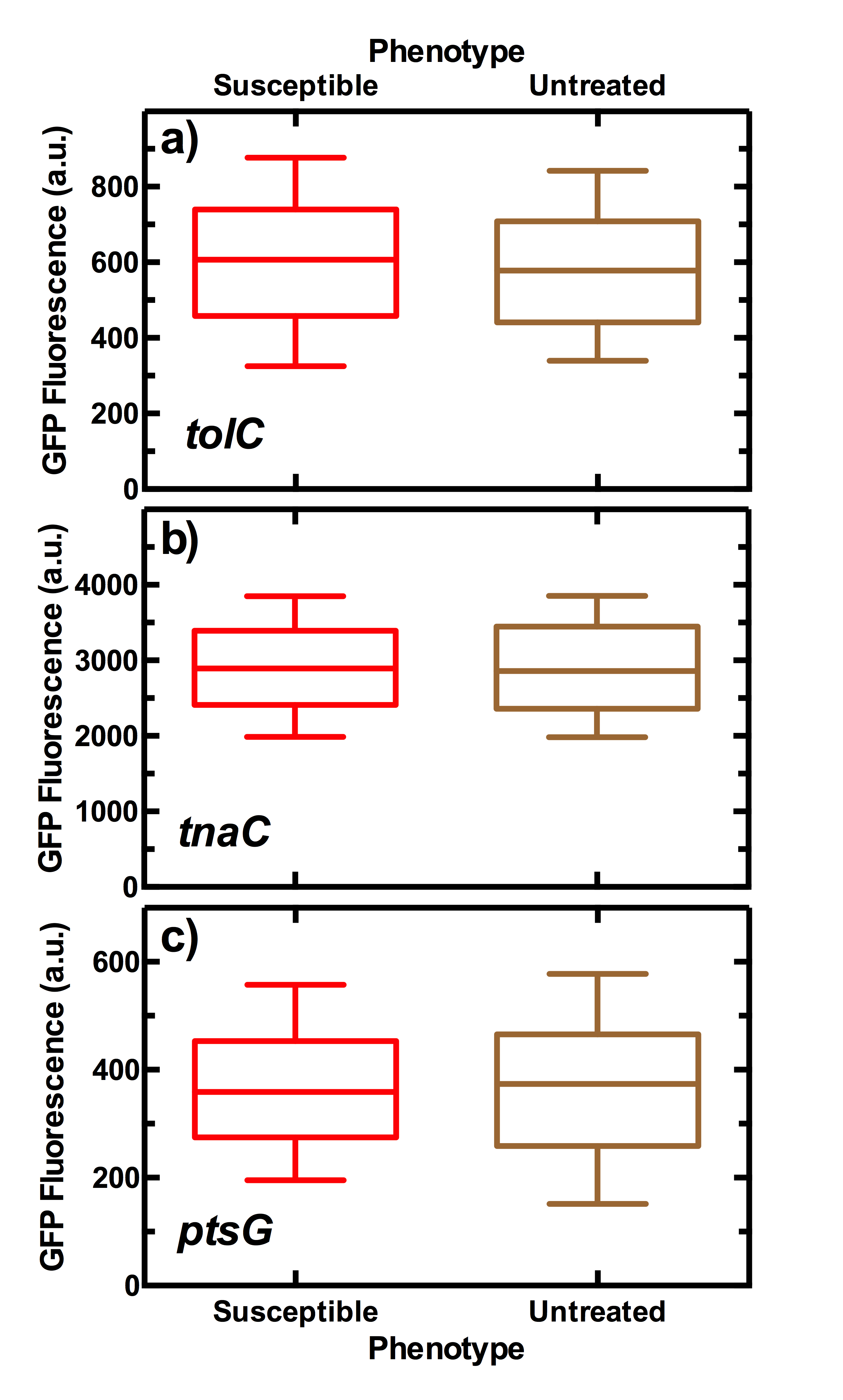

Supplement: Supplementary file 5 — Susceptible cells are indistinguishable from untreated cells before drug treatment. Distribution of fluorescence levels in the susceptible phenotype before drug treatment (t = 0) and in untreated control cells before regrowth in LB (t = 0) in the a) tolC, b) tnaC, and c) ptsG reporter strain. The two populations are not statistically different, an unpaired t test with Welch’s correction yielding a P value of 0.07, 0.7, and 0.9, respectively. The bottom and top of the box are the first and third quartiles, the band inside the box is the median, the bottom and top whiskers represent the 10th and 90th percentiles, respectively. Data are obtained at least in biological triplicate (N = 3) for each reporter strain employed for a total of n S = 6659 and n C = 3076 susceptible and control cells, respectively. We did not observe any significant difference between the results obtained from different biological replica. (PNG 423 kb) [file 12915_2017_465_MOESM5_ESM.png]

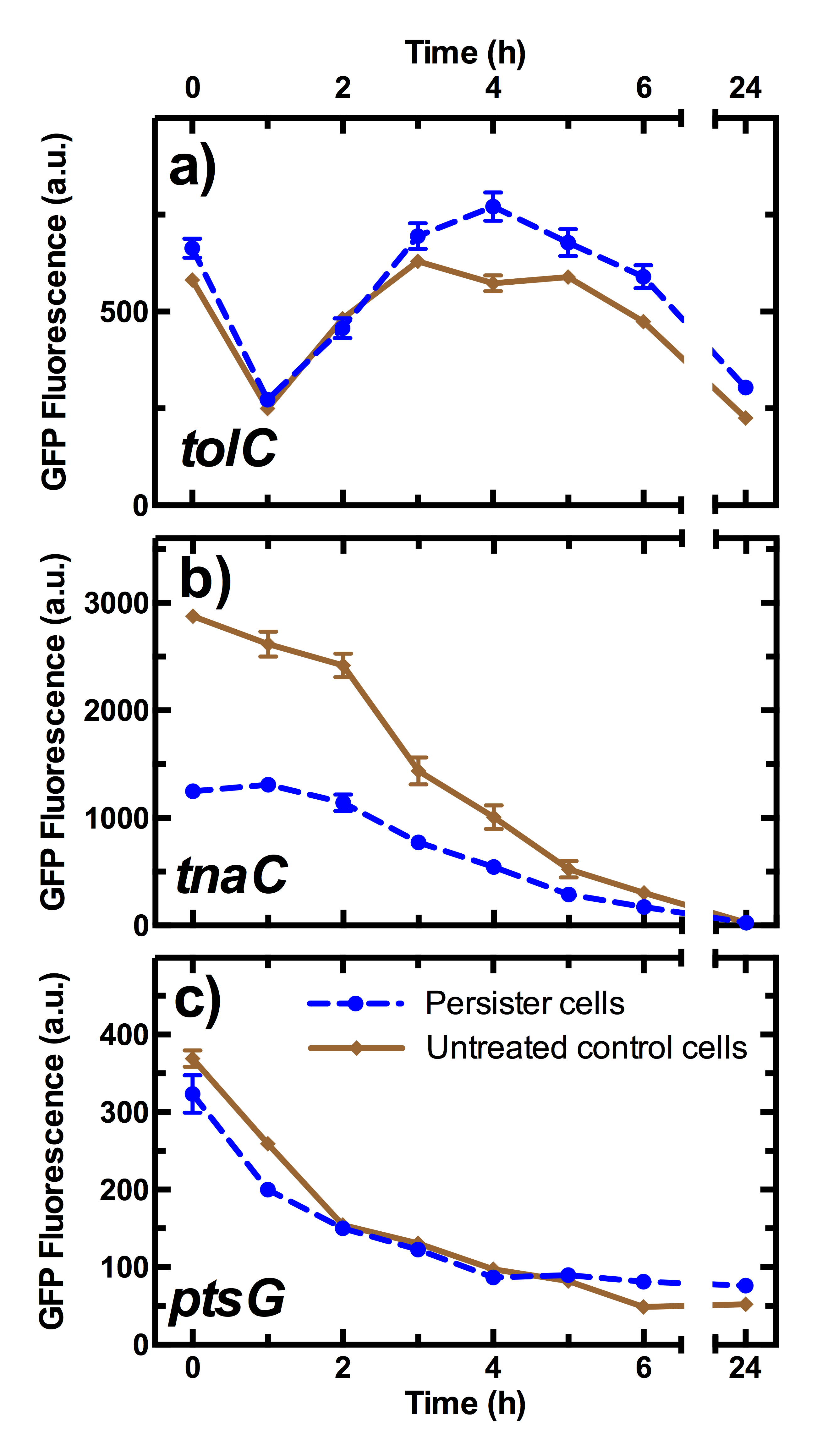

Supplement: Supplementary file 6 — Persister and untreated control cells have similar patterns of fluorescence levels during regrowth on LB. Average pattern of fluorescence levels in persister (circles) and untreated control (diamonds) cells throughout the microfluidic assay in the a) tolC, b) tnaC, and c) ptsG reporter strains. Persister and untreated control cells exhibit similar patterns of fluorescence levels during regrowth on LB (3 < t < 24 h and 0 < t < 24 h for persister and control cells, respectively) demonstrating that persister cells revert back to a normally growing state after removal of the antibiotic drug. Data and error bars are obtained as the mean and standard error of the mean of single-cell measurements in biological triplicate (N = 3) for each reporter strain for a total of n P = 132 and n C = 3076 persister and control cells, respectively. We did not observe any significant difference between the results obtained from different biological replica. Due to the large sample sizes, error bars are small compared to the corresponding mean values and are hidden behind the data points. (PNG 585 kb) [file 12915_2017_465_MOESM6_ESM.png]
